# 

**Published:** 1892-07-02

**Authors:** 


**WITH EXTRA SPECIAL SUPPLEMENT.**
THE REPORT ON METROPOLITAN HOSPITALS BY THE LORDS COMMITTEE.

### CONTENTS.

The Report of the Lords Committee . . . . . 209

Passing Topics.—Victory . . . . . . . 210

The Doctor's Armchair.—The Other Cheek . . . 211

The Attitude of Great Writers Towards Disease. IV.—Jane Austen . . . . . . . . 212

Medical History from the Earliest Times. By E. T. Withington, M.A., M.B.Oxon. XV.—Military Medicine in Ancient Greece . . . . . . 213

Everybody's Page . . . . . . . 214

Annotations.—Nurses and Long Skirts—Who of us is fit to Practise?—Tennis or Golf for Ladies? . . . . 215

Notes and News . . . . . . . . 216

Around the Hospitals—Scraps and Gleanings . . . 217

The Practitioners' Mirror and Retrospect—

Medicine.—Gangrene—The Position of the Medical Examiner for Life Assurance.—III. (*continued*) . . . 218

Therapeutics.—The Therapeutical Action of the Salts of Strontium . . . . . . . 219

Books Received . . . . . . . 220

The Institutional Workshop—

Hospital Administration. I.—Organisation . . . 221

Hospital Construction.—Paddington Infirmary . . . 222

Institution Literature.—Asylums of the World . . . 223

The Student of Medicine.—Appointments—Vacancies, &c.

**EXTRA SUPPLEMENT.—THE NURSING MIRROR.**

En Passant.—Royal South Hants Infirmary—The Registration of Midwives—He or She?—Private Nurses in Dublin—St. Kilda—Stockton and Thornaby District Nurses—Short Items—Allhallows, Ditchingham . . . . . xciii

Ventilation, Disinfection, and Diet. By P. Caldwell Smith, M.D. XII.—Diet and Dietaries . . . . xciv

The Lords' Report on the Proposed Registration of Nurses and British Nurses' Association . . . xcv

Colonies for Epileptics . . . . . . xcvi

The Toronto General Hospital Training School . . xcvi

For Reading to the Sick.—"Honest Doubt" . . . xcvii

The Hamilton Association for Providing Trained Male Nurses . . . . . . . xcviii

The Nurses and the Basuto War Medal . . . . xcviii

Presentation—Notes and Queries . . . . xcviii

Wants and Workers . . . . . . . xcviii

Small-Pox and Infectious Fever Nursing . . . xcix

Ring-Worm and Mob Caps . . . . . . xcix

The Metropolitan Provident Medical Association . xcix

Off Duty.—Four Months' in a Hospital Ward.—VII. . . . c

